# Identifying service quality gaps between patients and providers in a Native American outpatient clinic

**DOI:** 10.1186/s12913-022-07538-w

**Published:** 2022-02-08

**Authors:** Robert Dorsey, David Claudio, María A. Velázquez, Polly Petersen

**Affiliations:** 1 Department of Mechanical and Industrial Engineering, Montana State University, Bozeman, MT USA; 2 Department of Mechanical Engineering, University of Massachusetts Lowell, Lowell, MA USA; 3 College of Nursing, Montana State University, Bozeman, MT USA

**Keywords:** Native American, Outpatient clinic, Service Quality Gap, Expectations, Perceptions

## Abstract

**Background:**

Native American communities in Montana reservations have reported low-level satisfaction in health services. This research explored if the services provided at a Blackfeet Indian Reservation outpatient clinic were designed to meet patient expectations.

**Methods:**

Staff and patient interviews and surveys allowed service expectations to be assessed according to the clinic’s ability to meet those expectations. A total of 48 patients and ten staff members (83% of the staff at this clinic) participated in the study voluntarily.

**Results:**

We found a disconnect between what patients anticipate for care and what staff think they are anticipating. We also found a discontent between what staff believes patients need versus what the patients feel is needed.

**Conclusions:**

These gaps combine to increase the breach between patient expectations and perceptions of their healthcare services. With better insight that captures what patients are looking for from a service, the potential to meet those needs increases, and patients feel that their voice is respected and valued.

## Background

The ability to receive critical healthcare for rural areas is crucial as people live in isolated regions [1, 2]. What can be even more challenging is when much of the population suffers from economic poverty [3]. This reduces an individual’s ability to receive the valued healthcare that they need promptly.

Native American communities found in Montana reservations with the federally funded Indian Health Service (IHS) fall within the category of rural healthcare systems [4]. In many cases, low-level satisfaction results from a disconnect in what patients look for in the service compared to what a clinic has determined to be the appropriate service.

Since 2008, the Hospital Consumer Assessment of Healthcare Providers and Systems (HCAHPS) has offered a valid standard comparison tool for satisfaction criteria collection and reporting [5, 6]. Table 1 presents a CMS report comparing patient satisfaction with their healthcare on the Blackfeet Indian Reservation with the state of Montana and nationally [7].

**Table 1:** CMS Report - Blackfeet Healthcare [7]

	Hospital at Browning- Blackfeet (%)	Montana Average (%)	National Average (%)
Patients who reported that their nurses “Always” communicated well.	72	81	81
Patients who reported that their doctors “Always” communicated well.	79	83	82
Patients who reported that they “Always” received help as soon as they wanted.	72	75	70
Patients who reported that the staff “Always” explained about medicines before giving it to them.	54	69	66
Patients who reported that their room and bathroom were “Always” clean.	58	73	76
Patients who reported that the area around their room was “Always” quiet at night.	75	64	62
Patients who reported that YES, they were given information about what to do during their recovery at home.	70	86	87
Patients who “Strongly Agree” they understood their care when they left the hospital.	42	52	54
Patients who gave their hospital a rating of 9 or 10 on a scale from 0 (lowest) to 10 (highest).	52	70	73
Patients who reported YES, they would definitely recommend the hospital.	44	71	72

The report shows lower levels of satisfaction in the Blackfeet Nation than state and national averages. Communities such as the one on the Blackfeet Reservation can benefit from changes that could improve patient satisfaction and increase their health services’ quality.

**Fig. 1:**
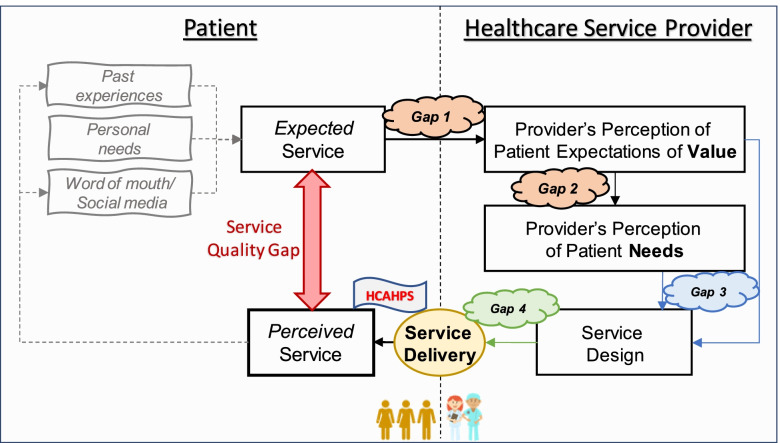
Conceptualization of the healthcare Service Quality Gap Model

Patient satisfaction or dissatisfaction is associated with the Service Quality Gap (SQG), which is the difference between what patients expect from a service and their perception of the service they receive [8, 9]. Figure 1 depicts the patient-provider interaction that could lead to SQG. The figure is based on the Service Quality Gap model [10, 11].

The SQG model starts with a patient having a concern or a need. The patient then chooses a healthcare provider according to past experiences, word of mouth, or simply because that is the only provider available [12–15].

According to the SQG model, there are many opportunities in which gaps or divergences could occur. For instance, Gap 1 focuses on a misalignment between patients’ expectations from the service and what the provider thinks patients expect. Gap 2 identifies what healthcare providers think patients expect versus what they think patients need, which could differ in their expert opinion.

The Design Gap (Gap 3) focuses on how staffing, operations, processes, layout, and patient and information flow are designed to provide the best healthcare service possible. It also includes the physical surroundings, ambient, decorations, and cleanliness of the location. The Service Delivery Gap or Service Encounter Gap (Gap 4) is related to the human resources aspect, such as friendliness, responsiveness, empathy, inclusivity, and employee thoughtfulness.

These four gaps combine to influence patients’ service experiences and their perception of the quality of a service [16–18]. The difference between expected service and the perception of the service received creates the Service Quality Gap. Therefore, minimizing the first four gaps can increase patients’ satisfaction levels at a facility while still allowing healthcare staff to provide the needed services [17]. By doing so, there is potential for improvement of the patient’s experience in their local clinics. This, in turn, could improve the satisfaction rating of the facility, which is standardized and reported for public use, accreditation, and reimbursement purposes [19].

The literature shows evidence of healthcare providers using HCAHPS or other types of surveys to improve their customer service [20–27]. However, many of the reported cases appear to react to survey results as improvements are made only after services have been provided and measured.

In retrospect, this approach can still leave a SQG that needs to be addressed and service design can take a long time to align with patient expectations. With the ability to bring forward what each party values in their services, the ability to meet needs and expectations satisfactorily becomes more viable prior to the visit and leads to a proactive approach.

On the other hand, relying too heavily on satisfaction surveys could lead to poor healthcare practices since providers would be focusing too much on what patients want to achieve higher scores [28]. This implies that “patient wants” need to be considered but only concerning what the healthcare staff can do to treat patients effectively (patient wants vs. patient needs; Gap 2).

The balance between designing for patients’ expectations versus effective care can be difficult to assess through post-service surveys such as HCAHPS. It is important to include patient feedback when designing or re-designing a process. Baker [29] maintains that patients want to be part of the healthcare process; listening to their voice before they receive service is an important dimension of a Patient-and Family-Centered Care (PFCC) approach to healthcare design and improvement [30, 31]. In fact, it is one of the eight dimensions of PFCC [31], which is essential to any health provider, but in particular, for those in isolated regions [1] like the one on the Blackfeet Indian Reservation. From the results presented in Table 1, it was important to investigate why the Native American community in Browning, Montana has lower HCAHPS scores than state and nation averages.

Weidmer-Ocampo et al. [32] adapted CAHPS and surveyed a Native American population in Oklahoma. Interviews were conducted with a small group of patients to ensure the survey’s cognitive understanding was developed. Afterward, the survey was distributed via mail one week after their visit to assess their satisfaction with the healthcare facility. Their results were successful in providing meaningful direction to improve patient satisfaction with the services. While Weidmer-Ocampo et al. [32] assessed a Native American population, service expectations were not assessed prior to the visit to allow patients to have a voice in the re-design or improvement process. It was assumed that the CAHPS assessed patient expectations.

This research study explored if the services provided at a Blackfeet outpatient clinic are designed to care for the patient and meet the expectations patients anticipate. The research focused on the first two gaps of the SQG model to uncover potential misalignments between patient and healthcare provider service expectations in the Blackfeet Indian Reservation clinic. Staff and patient interviews and surveys allowed service expectations to be assessed according to the clinic’s ability to meet those expectations.

## Methods

Before starting the research, Institutional Review Board approvals from Montana State University, Indian Health Service (IHS), and the Blackfeet Tribal Council were obtained.

### Setting

The outpatient clinic in which the study took place has 16 physicians and covers ten specialty areas of medicine. It is located in the Blackfeet Indian Reservation in Browning, MT, and is home to over 7000 descendants of the Ampska Pikuni Nation.

Individuals who want an appointment for the day start out by either making a call or being at the clinic at 6 a.m. When the call is made, patients wait for the secretary or nursing assistant on shift to answer the call. Patients are asked if they would prefer to see a specific physician or if they have a preference on the time of day to be seen. This is all dependent on patients being able to call or present themselves early enough to obtain an appointment, which has been an issue in all departments within this facility. Once patients obtain an appointment, they are asked about symptoms and then assigned a time to check-in for the appointment.

Arriving at the facility, patients check-in, during which time is spent updating contact information. After this process, patients sit in the waiting area near the clinic. Patients wait there until a nursing assistant calls them into the clinic. A nursing assistant collects patients’ vitals and reconfirms the health complaint. From here, patients are brought to an exam room where they wait for the physician to arrive and see the patients for any health issues.

In both the waiting area and in the exam room, the waiting time that occurs from entering the facility to be seen by the physician is approximately one hour. During the visit, the physician can have additional lab work or x-rays ordered to further investigate the health issues or patient complaints. Upon completing the visit with the provider, patients can leave or wait for medications that may have been prescribed.

### Participants

Participants included patients and staff. Participation in the research was voluntary, and participants could stop at any moment. Participants were not incentivized for their participation. All responses for patients and staff were kept anonymous, and results were reported at an aggregate level.

Patients were selected randomly during a week of normal business in November 2019. One of the researchers, who is also part of the Blackfeet Nation, approached patients 18 years of age or older who had just arrived at the clinic and had to wait for their visit with the provider. The researcher explained the study and obtained written informed consent before doing the questionnaire. When the researcher completed one survey, he recruited the next patient by looking for patients that had just arrived.

Staff was composed of registered nurses (RN), certified nursing assistants (CNA), and administrative staff who worked at the Outpatient Clinic on the same week data was collected. We excluded pediatric staff due to the scope of the research (adult patient population). Given the small number of staff at the clinic, questions such as “years of experience” or “years working at this location” were excluded as they could have been potential personal identifiers.

### Survey

A survey was developed in Qualtrics. The survey was composed of three major categories: demographic data, open-ended questions, and a Multiple-choice Likert scale questionnaire. Staff only answered the first two parts (demographics and open-ended questions). Patients completed all three parts of the survey. The demographic data included age, gender, marital status, and employment status. The open-ended questions were:

1. *What do you look for in your healthcare service?*
2. *What do you expect from your healthcare provider?*
3. *What didn’t you like from previous visits to this facility?*
4. *What is not important to you during your service visit?*

**Fig. 2:**
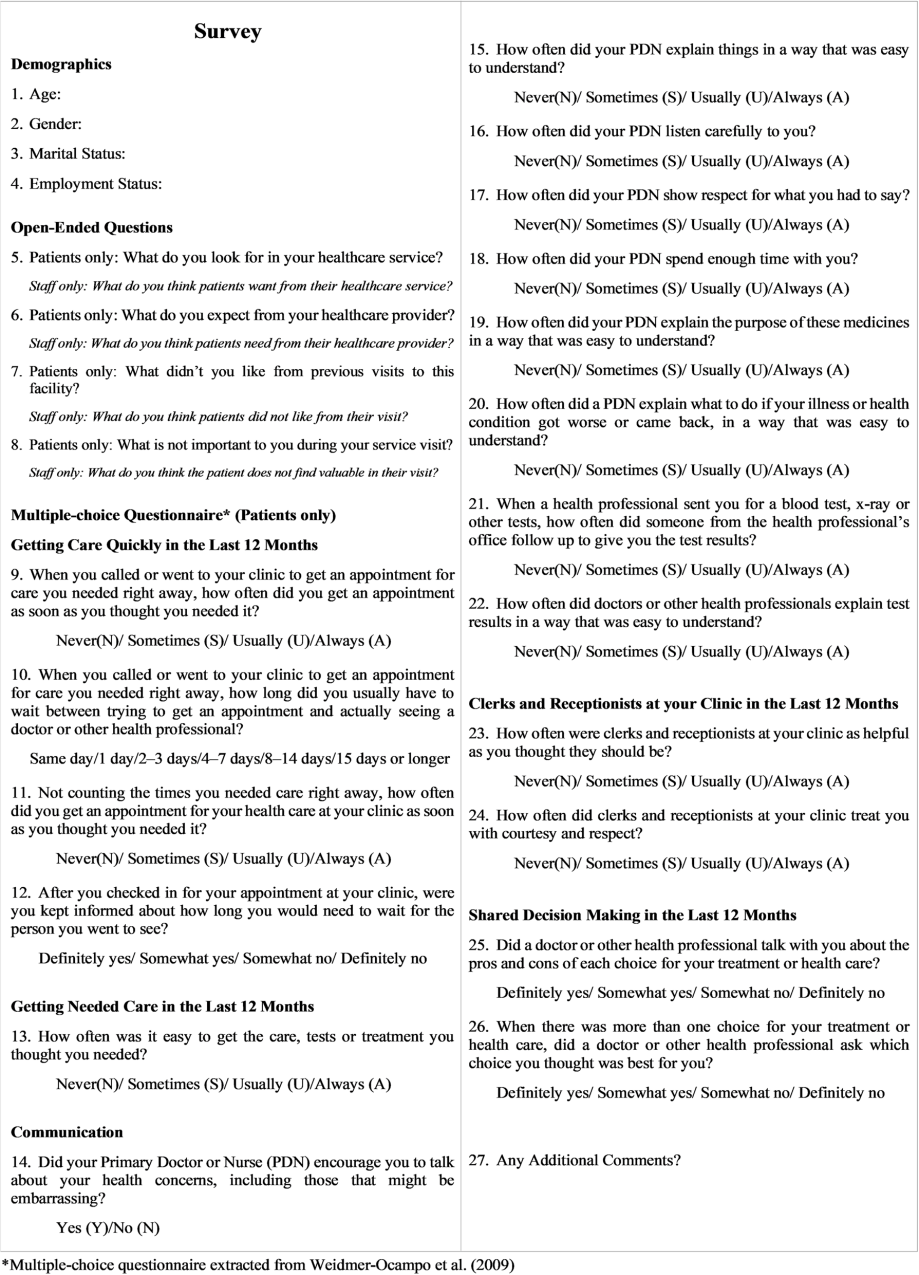
Research Study Survey

The multiple-choice Likert scale questionnaire was adopted from a previously published article from Weidmer-Ocampo et al. [32] with established validity. The questions were placed in Qualtrics survey software for faster analytics on patients’ views of the clinic’s performance. The items were scored with 4-point Likert Scale reasoning for each question’s performance, 1 being best and 4 worst. Figure 2 presents the survey.

### Data Collection

Survey administration occurred during regular operating hours for a week in November 2019. The interviews were done with all the staff and a random selection of patients that volunteered to participate. Through collection on both sides of the process, separation between the two groups was maintained.

Patient interviews were conducted by a researcher who is also part of the Blackfeet Nation. This allowed insight that brings together technical and personable aspects for the entire process for patient satisfaction improvement. The researcher explained the study to potential participants. Upon obtaining written informed consent to participate in the survey, the researcher took the participants into a private room near the waiting area. The researcher had the entire survey (Fig. 2) uploaded in Qualtrics on his tablet. He would begin the process by handing over the tablet to the participants and asking them to answer the first four multiple-choice demographic questions.

The researcher would then take the tablet and ask the four open-ended questions, one at a time. He typed the participants’ responses in Qualtrics on the tablet via a Bluetooth keyboard. This process allowed participants to have a casual conversation while the researcher recorded the answers. The researcher did not ask additional questions or prompt additional information. Upon completing the four open-ended questions, the researcher handed the tablet back to the participants and asked them to complete the Multiple-choice Questionnaire.

Staff interviews occurred before the clinic opened, during lunch breaks, and after-hours so that regular operations were not interrupted. The researcher had the first two parts of the survey (demographics and open-ended questions for staff) uploaded in Qualtrics on his tablet. The researcher explained the study and obtained informed consent before handing over the tablet to the staff. Staff participants completed the first four multiple-choice demographic questions.

The researcher would then take the tablet and ask the four open-ended questions designed for staff only, one at a time. He typed the staffs’ responses in Qualtrics on the tablet via a Bluetooth keyboard. This process allowed staff to have a casual conversation while the researcher recorded the answers. The researcher did not ask additional questions or prompt additional information. Upon completing the four open-ended questions, the survey was done for staff as they were not asked to complete the Multiple-choice Questionnaire.

### Data Analysis

Demographic questions were separated by patients or staff and aggregated for each group. Statistical analysis was generated using Stats IQ™ from Qualtrics and presented at the aggregate level.

Responses to open-ended questions were also separated between patients and staff. Content analysis [33] was performed to categorize similar words within the interview context and count the frequency of the words. A word cloud was created for each open-ended question with those words entered most frequently appearing largest in the word cloud.

The open-ended question *“What is not important to you during your service visit?”* (Question #4) had a vast majority answered in the opposite, stating items that they found important or that everything is important. That information was unusable due to the type of responses as we could not obtain the information we were expecting with this question. It should be noted that even though we thought people were going to find it easier to identify what they didn’t think was important, a point is to be made about not asking questions in the negative sense in the future for what is important in healthcare.

Statistical analysis for the Multiple-choice Questionnaire was also generated using Stats IQ™ from Qualtrics and presented at the aggregate level for each question.

## Results

A total of 48 patients and ten staff members for the designated clinic participated in the study voluntarily. The staff was composed of three registered nurses, four clinical nurse assistants, and three clerical staff. Survey administration occurred during regular operating hours over a week in November of 2019 (pre-COVID-19). All responses for patients and staff were kept anonymous, and results were reported at an aggregate level for each group.

Table 2 presents a summary of the demographics for patients and staff. Of both groups of participants, most were female (75.86%). Age had a normal distribution between ages 21 to 72. Most of the patient participants were employed full-time at 60.42%. The staff response rate was strictly from staff working on the same week data was collected, and they only answered the demographic and the open-ended questions. The total response rate accounted for 83% of the staff in this specific clinic.

**Table 2:** Demographics for patients and staff

Age Group	Patients	Staff
18–24	2	0
25–34	5	1
35–44	12	4
45–54	9	4
55–64	12	1
65 and older	8	0
Gender		
Female	34	10
Male	14	0
Employment Status		
Employed full time	29	10
Employed part-time	2	0
Unemployed (looking for work)	7	0
Unemployed (not looking for work)	1	0
Retired	5	0
Student	1	0
Disabled	3	0

### Open-ended Questions for Patients

Some issues that patients voiced but were not recorded during the survey include an unprofessional demeanour, feeling as if they are “a burden to the staff,” and lack of available appointments. These concerns were reported to the providers when we presented the result of the study to them.

**Fig. 3:**
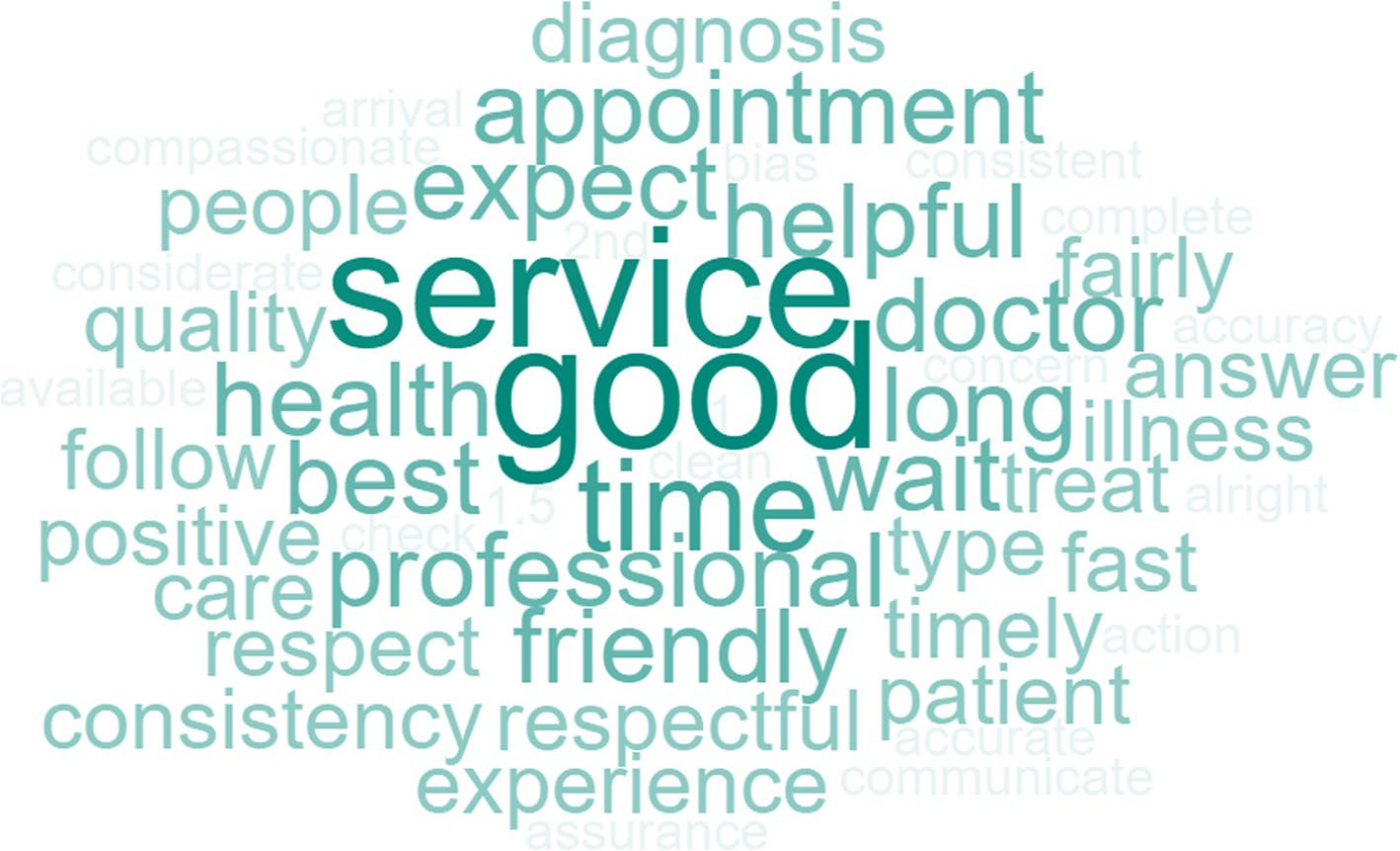
Patient Word Cloud for “What do you look for in your healthcare service?”

Responses for question 1 of the patient survey, *“What do you look for in your healthcare service?”* had three responses at the top that included *quality or good visit*, *respect*, and *on-time*. The frequency of word responses is shown in Fig. 3. Other responses included *thorough diagnosis*, *availability of appointments*, and *set protocol*. The other responses show that patients want a comprehensive visit for their ailments, want *to get an appointment as needed,* and *follow steps consistently throughout the process*.

**Fig. 4:**
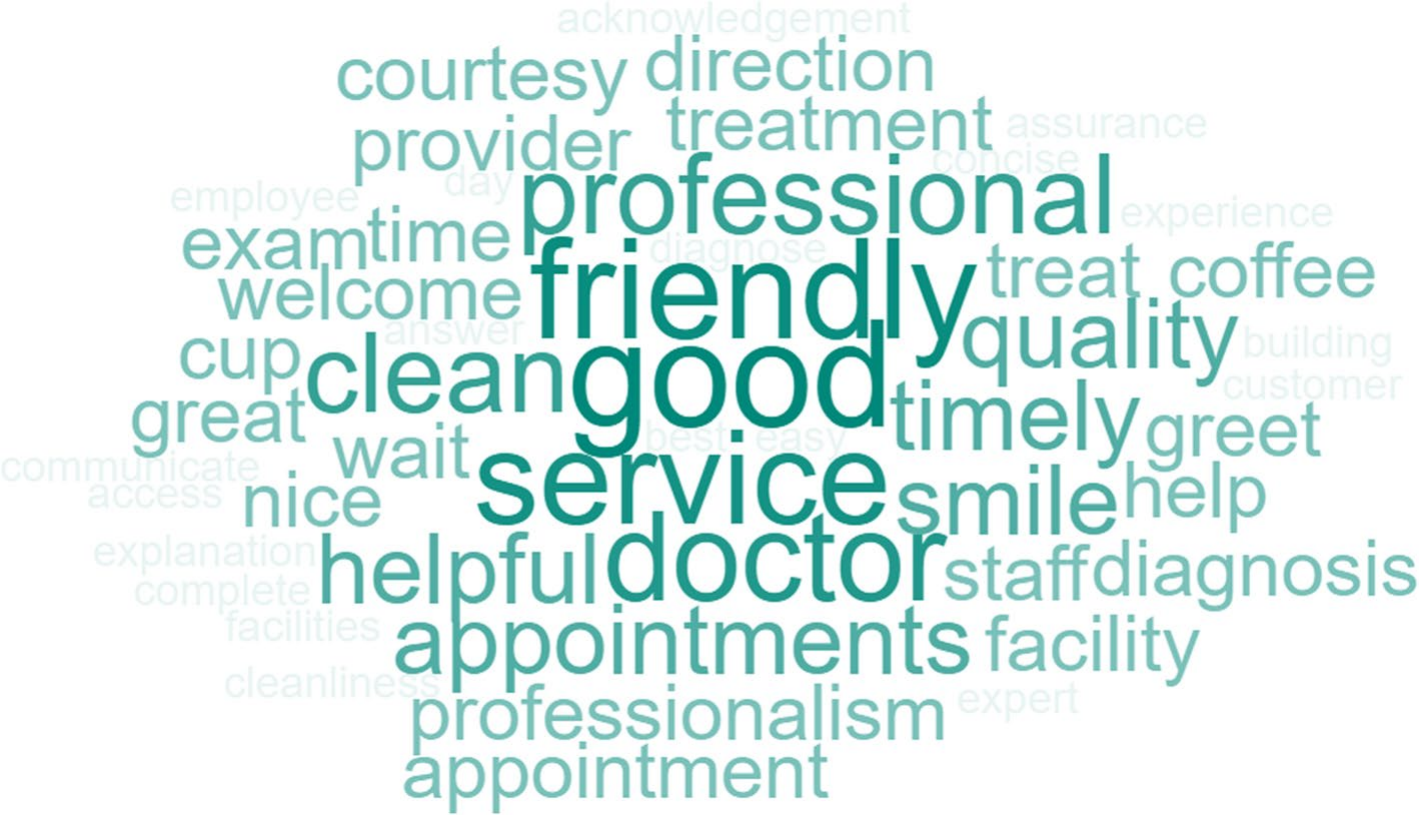
Patient Word Cloud for “What do you expect from your healthcare provider?”

For question 2 of the patient survey, *“What do you expect from your healthcare provider?”* the top three, in order, were *customer service*, *thorough diagnosis*, and *professionalism*. Other responses also included a *clean facility*, *prompt service*, and *clear communication*. The frequency of word responses is shown in Fig. 4.

**Fig. 5:**
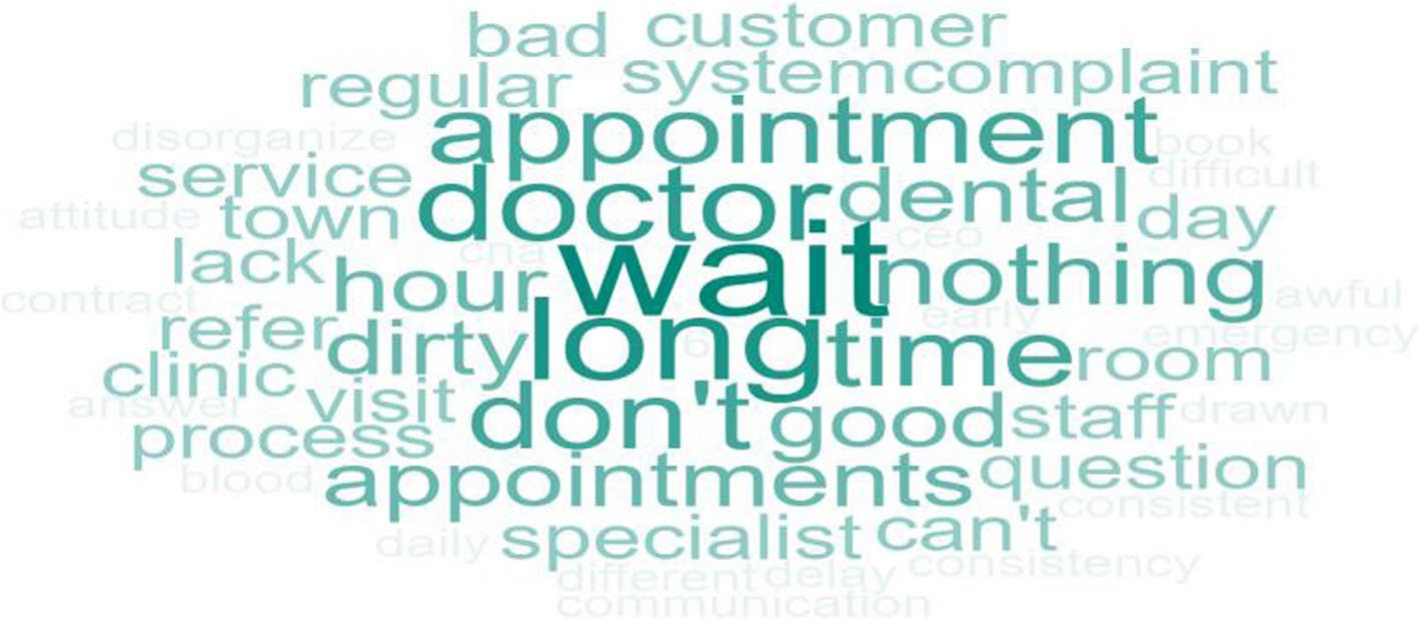
Patient Word Cloud for “What didn’t you like from previous visits to this facility?”

Question 3 of the patient survey explored what patients did not like about the service (*“What didn’t you like from previous visits to this facility?”*). The top three responses were *wait time*, *lack of regular appointments*, and *poor customer service*. Wait time includes the time in the waiting area and waiting in the exam room to be seen by the provider. Poor customer service includes rude behavior and feeling like a burden to staff. Other responses included *cleanliness* and *lack of explanation*. The frequency of word responses is shown in Fig. 5.

### Open-ended Questions for Providers

**Fig. 6:**
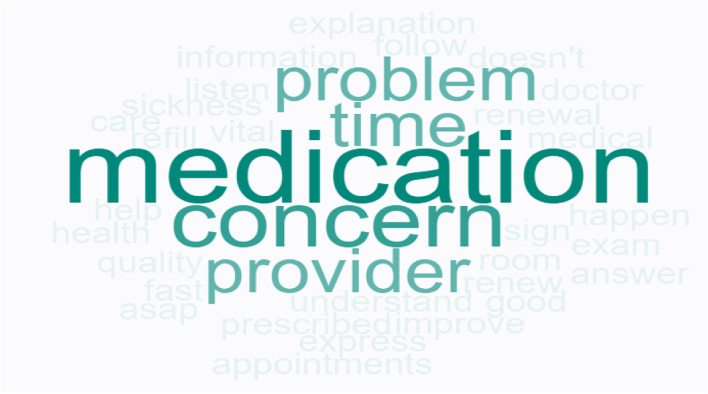
Staff Word Cloud “What do you think patients want from their healthcare service?”

Question 1 for the healthcare staff, *“What do you think patients want from their healthcare service?”* had the following top responses*: medication refill* and *good doctor*. Other responses include *information to improve health*, *thorough visit*, and *time (in system or with doctor)*. The frequency of word responses is shown in Fig. 6.

**Fig. 7:**
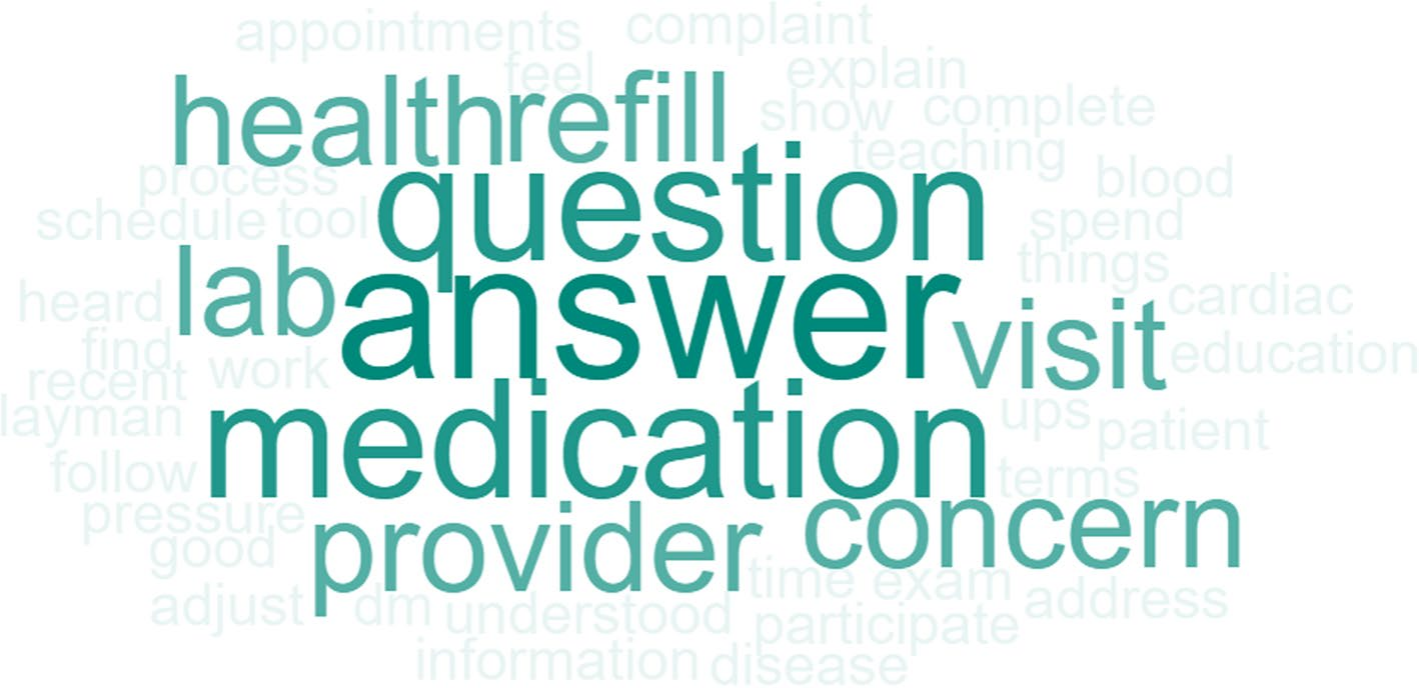
Staff Word Cloud “What do you think patients need from their healthcare provider?”

Question 2 for the healthcare staff, *“What do you think patients need from their healthcare provider?”* had the top two responses of *communication (information and tools to improve health)* and *participation during visit*. Other responses included a *thorough examination* and *health education*. The frequency of word responses is shown in Fig. 7.

**Fig. 8:**
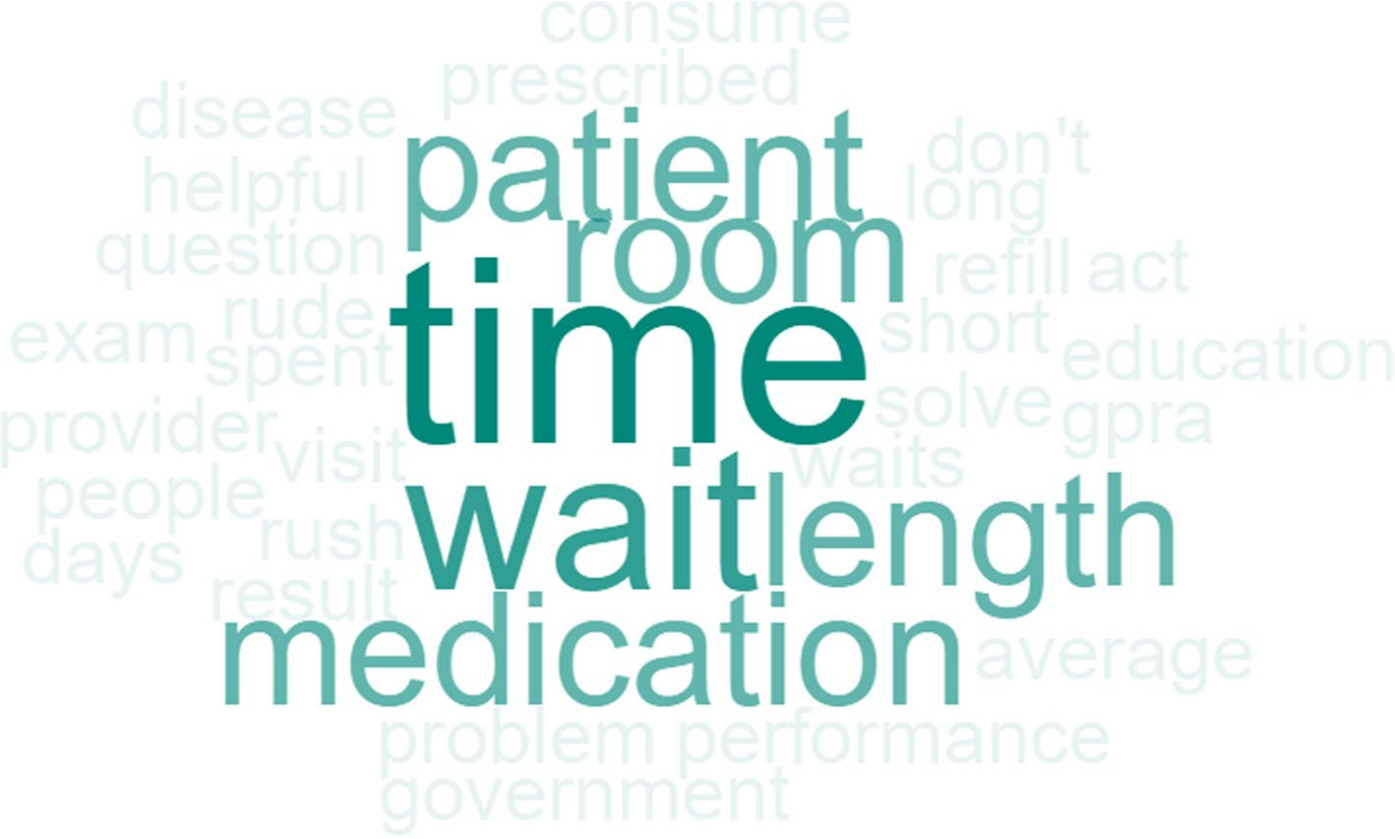
Staff Word Cloud “What do you think patients did not like from their visit?”

Question 3 for the healthcare staff was, *“What do you think patients did not like from their visit?”.* The top two responses for this were *long wait*s and *short time with provider*. Other responses included *rudeness* and *rushed*. The frequency of word responses is shown in Fig. 8.

### Multiple Choice Questionnaire (Patients only)

The set-up of questions in the Qualtrics resulted in lower scores representing better satisfaction which means that having a score closer to 1 is better performing than a score closer to 4. For example, for question 10, a mean score of 2.74 is on the scale’s negative side. A score of 1 would represent that all patients felt they got appointments as soon as they needed them. Question 15 had a yes or no response, which is the cause of a max number of 2. Question 11 has a maximum value of 6 due to the number of potential responses, with a higher value still meaning worse performance. Additionally, questions 17, 18, and 19 have a max of 3 because no one answered the “Never” response. Table 3 presents the aggregated results from the patient questionnaire.

**Table 3:** Patient Multiple-Choice Questionnaire Statistics

Patient questions	Min Score	Max Score	Mean Score (Performance %)	Std. Deviation
Q09- When you called or went to your clinic to get an appointment for care you needed right away, how often did you get an appointment as soon as you thought you needed it?	1.00	4.00	2.74 (73.27%)	0.87
Q10- When you called or went to your clinic to get an appointment for care you needed right away, how long did you usually have to wait between trying to get an appointment and actually seeing a doctor or other health professional?	1.00	6.00	3.33 (53.40%)	1.48
Q11- Not counting the times you needed care right away, how often did you get an appointment for your health care at your clinic as soon as you thought you needed it?	1.00	4.00	2.43 (52.81%)	0.82
Q12- After you checked in for your appointment at your clinic, were you kept informed about how long you would need to wait for the person you went to see?	1.00	4.00	2.75 (42.25%)	0.90
Q13- How often was it easy to get the care, tests or treatment you thought you needed?	1.00	4.00	2.38 (54.46%)	0.93
Q14- Did your Primary Doctor or Nurse (PDN) encourage you to talk about your health concerns, including those that might be embarrassing?	1.00	2.00	1.29 (71.00%)	0.45
Q15- How often did your PDN explain things in a way that was easy to understand?	1.00	4.00	2.21 (60.67%)	0.96
Q16- How often did your PDN listen carefully to you?	1.00	4.00	1.92 (69.64%)	0.73
Q17- How often did your PDN show respect for what you had to say?	1.00	4.00	1.81 (73.27%)	0.75
Q18- How often did your PDN spend enough time with you?	1.00	4.00	2.17 (61.39%)	0.75
Q19- How often did your PDN explain the purpose of these medicines in a way that was easy to understand?	1.00	4.00	2.21 (60.07%)	1.04
Q20- How often did a PDN explain what to do if your illness or health condition got worse or came back, in a way that was easy to understand?	1.00	4.00	2.13 (62.17%)	0.99
Q21- When a health professional sent you for a blood test, x-ray or other test, how often did someone from the health professional’s office follow up to give you the test results?	1.00	4.00	2.73 (42.91%)	1.15
Q22- How often did doctors or other health professionals explain test results in a way that was easy to understand?	1.00	4.00	2.26 (58.42%)	0.98
Q23- How often were clerks and receptionists at your clinic as helpful as you thought they should be?	1.00	4.00	2.19 (60.73%)	0.99
Q24- How often did clerks and receptionists at your clinic treat you with courtesy and respect?	1.00	4.00	1.92 (69.64%)	0.89
Q25- Did a doctor or other health professional talk with you about the pros and cons of each choice for your treatment or health care?	1.00	4.00	2.24 (59.08%)	0.81
Q26- When there was more than one choice for your treatment or health care, did a doctor or other health professional ask which choice you thought was best for you?	1.00	4.00	2.20 (60.40%)	0.85

Patients responded that they did not get an appointment as soon as they felt they needed to be seen. They also felt there was a lack of feedback or follow-up regarding x-ray or lab results. This is determined from the higher mean scores of these multiple-choice items as the higher the mean score, the lower that item was rated on its performance. A score of 1.75 is related to a performance score of 75%, a score of 2.50 is related to a performance of 50%, and a score of 3.25 is related to a performance of 25%.

Most items fell between a rating of 50 and 75%. Results were consistent with Weidmer-Ocampo et al. [32], which showed that getting care quickly and the Clerks/Receptionists interactions were rated higher than other constructs.

## Discussion

### Comparison of answers to open-ended questions

We will now compare the responses obtained from open-ended questions from patients against responses obtained from staff for similar questions to uncover potential service gaps in the system.

### What patients look for vs. what staff think patients want (Gap 1)

Comparing what patients look for in their healthcare and what staff thinks patients want in their healthcare (Q1 patients vs. staff) identifies shortcomings in Gap 1 of the SQG model. From the information collected, patients look for quality care, respect, and timely care. The staff’s top two responses were patients who wanted medication refills and a quality physician. In this simple side-by-side comparison, differences or gaps are already identified.

The biggest difference is that patients expect to be respected and receive timely care, whereas staff thinks patients are there merely for medication refills. The second difference is a little more subtle; while patients want quality care, the staff thinks that they only care about the quality of the physician. The difference here is that staff do not consider their interaction with patients as part of the patient’s healthcare experience. In contrast, patients look for an overall quality experience from the moment they enter the clinic. We believe that, at this clinic, staff might not be fully aware of the importance and magnitude their attitudes and behaviors have on the overall patient experience. Results show a disconnect between what patients are looking for in their service and what staff thinks patients want.

Our results align with Ostrov, Reynolds, and Scalzi [34], who assessed patient satisfaction between two healthcare units. Like our study, their questionnaire included what the physicians and nurses believed patients wanted. The survey found that the service the patients preferred was not the same service staff had thought would be preferred.

### What staff think patients want vs. what staff think patients need (Gap 2)

Comparing what staff thinks patients want from their healthcare service and what staff thinks patients need from their healthcare provider (*Q1 vs. Q2 for staff)* aims to identify shortcomings in Gap 2 of the SQG model.

The staff thinks what patients want from their healthcare are medication refills and a quality physician from the information collected. The staff’s top two responses for patient needs were information communication and acknowledgment during the visit. In this side-by-side comparison, we can again identify the difference between what staff thinks patients look for in their service and what they think patients need. In this case, staff believes patients need to “hear and be heard,” as one of the staff members stated.

The answers from staff about patient needs are not surprising as *“information and education”* and “*participation*” are two core principles of PFCC [35]. Acknowledgment or participation might still be related to quality physician, but it goes beyond the patient-physician interaction. Responses indicate that at this particular facility, the staff is still missing awareness of “Collaboration” and “Dignity and Respect,” which are also core principles of PFCC [35].

### What patients look for vs. what staff think patients need (Gap 1 + Gap 2)

A third comparison is between what patients look for and what the staff thinks patients need from the healthcare visit (Q1 patients vs. Q2 staff). This comparison aims to identify any shortcomings in Gaps 1 and 2 in the SQG. Once again, from the information collected, patients look for quality care, respect, and timely care. The staff’s top responses for what they think the patient needs were the communication of information and acknowledged participation of the patient during their visit.

Results indicate a disconnect between what patients look for and what staff think they are looking for (Gap 1) and what staff believes patients look for versus what they need (Gap 2). If services are designed according to what providers believe patients need, there is still a disconnect from patient expectations. The Collaboration aspect of PFCC suggests patients, families, and healthcare providers “*collaborate in policy and program development, implementation, and assessment; in health care facility design; and in professional education, and in the delivery of care*” [35]. The disconnect shown in Gaps 1 and 2 can lead to Gap 3 when healthcare providers design their services without patient input. All these gaps combine to increase the breach between patient expectations and perceptions of healthcare services.

### What patients expect vs. what staff think patients need (Gap 1 + 2)

The comparison between what patients expect from their healthcare provider and what staff thinks patients need from their healthcare provider (Q2 patients vs. staff) aims to identify, once again, shortcomings in Gaps 1 and 2 of the SQG. From the information collected, the patient’s expectations from their healthcare include good customer service, a thorough diagnosis, and professionalism. The staff’s top responses for what they think patient needs were communication of information and acknowledged patient participation during their visit. Communication and acknowledgment are key components of good customer service, but other aspects, such as respect and empathy, make *customer service* a broader category. The expectation of a thorough analysis and professionalism were not considered by the staff. Ungureanu and Mocean [14] found that education, patience, and respect constitute a significant portion of what patients look for in their health service. Similarly, we find that patients look for respect and patience. Staff at this location agree on the importance of communication and information dissemination.

### What patients look for vs. what patients expect (Expected Service)

Comparisons of what patients look for in the healthcare service versus what they expect from the provider (Q1 vs. Q2 patients) were done to study patients’ potential conditioning or bias. We were interested in uncovering if patients were setting their expectations differently from what they were anticipating. From the information collected, patients look for quality care, being respected, and timely care. Additional expectations were good customer service, a thorough diagnosis, and professionalism. Good customer service and thorough diagnosis are related to quality care and being respected is related to professionalism. Interestingly, even though patients look for timely care, they did not expect it. This was consistent with the results from question 3.

### What patients didn’t like vs. what staff think patients did not like (Gap1)

Comparing what patients did not like and what staff thinks patients did not like (Q3 patients vs. staff) returns to explore Gap 1 further. From the information collected, the staff thinks patients do not like short visits with the provider or waiting to get an appointment. Other responses included not getting medications refilled, answering *Government Performance and Results Act* questions, and lack of explanation in medication, health education, and steps to improve health. Patients responded to this question with many stating the wait to get an appointment was a major dislike. The next two items that presented themselves were feeling mistreated and the wait to be seen. The two wait items differentiate because the former is an attempt to get an appointment and get in the system, while the latter is related to having obtained an appointment but waiting within the system to be seen by the provider. Both groups show that waiting to get an appointment is a dislike for people attempting to be seen. The staff’s first response of a short time with the provider did show up in one patient’s response. With many other items appearing more frequently than the staff’s top response, this appears not to be as important to the patient. A more significant item is the social treatment the patient receives.

### Multiple-Choice questionnaire (Patients Only)

The multiple-choice section shows that there is room for improvement, particularly the appointment process, which is an issue for patients; however, many items came back rating slightly higher than average. This indicates there is room to improve as many areas were not close to the exceptional level.

Questions that showed poor performance were related to getting an appointment and lack of feedback or follow-up regarding x-ray or lab results. In both the Weidmer-Ocampo et al. [32] study and this study, individuals valued a high level of care and respect while being seen in their healthcare facility. In both studies, we can see a difference in what patients expect from their healthcare than those of the healthcare staff.

We also found that the HCAHPS constructs created by Weidmer-Ocampo et al. [32] aligned with the open question concerning patient expectations. The HCAHPS constructs, which assess service delivery against patients’ perceptions of the received services, align with the service expectations. In that case, it can then be implied that low HCAHPS scores are due to one or more of the four gaps previously discussed.

This study showed the existence of Gaps 1 and 2 in the SQG model at a Native American healthcare clinic. However, low HCAPHS scores at the Blackfeet reservation result from a compounding effect of the two gaps discussed and the Design gap (Gap 3) and Delivery Gap (Gap 4). Hyde and Hardy [35] argue there is a lack of shared understanding and communication regarding what PFCC means and how it is experienced from the patient perspective.

## Conclusions

This study explored potential reasons why a clinic in a Native American reservation is receiving lower patient satisfaction scores in comparison to state and nation averages. Identifying reasons for lower performance will differ for different clinics and facilities; therefore, these results are not generalizable but still allow for the basic structure to ascertain similar issues elsewhere. The study explored if the services provided are designed to care for the patient and meet the expectations patients anticipate.

Addressing findings from the open-ended questions, there existed a clear distinction between what patients look for in their healthcare service versus what the staff had thought patients were anticipating. There had been a clear distinction that patients wanted or valued items that involved their treatment and care in the system. The staff response was directed more towards a result, such as the medication item. This has the possibility of bridging an area of difference in expectations. With this disconnect in expectations, the service provided might influence higher ratings in patient satisfaction. The ability to explore and assess any service value gaps further could bring to light the root issue. In doing so, effective corrective actions can be taken to address these differences.

At this particular clinic, patients look for respect and patience from the moment they enter the clinic. In contrast, some staff does not consider their interaction with patients as part of their healthcare experience. We believe that, at this clinic, staff might not be fully aware of the importance and magnitude their attitudes and behaviors have on the overall patient experience. Specific recommendations for this clinic include:

1. Improve staff active listening, communication, and respect for the patients. This could be achieved through improved awareness of their role in the patient experience and through customer service training.
2. Staff should make no assumptions on why patients are there.
3. Staff should provide timely feedback on labs and x-rays.
4. If the system cannot increase capacity to accommodate patient demand, staff should be able to explain at least why there might not be appointments sooner.

The third and fourth recommendations are closely related to the Design Gap (Gap 3), which is the one that focuses on how staffing, operations, processes, layout, and patient and information flow are designed to provide the best healthcare service possible. Further research is needed to assess the Service Design and Service Delivery Gaps (Gaps 3 and 4 respectively) at this clinic to uncover potential barriers to achieving patients’ expected outcomes. For example, the clinic might not have a clear policy on who is responsible for calling patients with labs and x-rays results, or they might have difficulty reaching patients. Perhaps they might have staffing issues, or the solution might lie in communicating reasonable expectations with patients (i.e., “a nurse will call in 1-2 weeks with the results, sooner if results are more urgent”).

With better insight that captures what patients are looking for from a service, as with any service industry, the potential to meet those needs better increases. Instead of being reactive in the improvement process, the aim will be proactive to enhance the patient experience and meet their needs. Understanding that some items may be of more value than others, contradicting previous thought and training, professionals can focus their critical time on what their customers value, particularly the patient.

In the case of healthcare facilities such as the clinic in this study, improved patient satisfaction with the service will support patient retention for providers employed in the clinic and not seek services elsewhere, resulting in increased reimbursement from CMS accreditation. It also creates an environment where patients feel that their voice is more valued, enhancing the feeling that the patient is respected.

This study is not without limitations. The biggest shortcoming is that the study was conducted at a single clinic within one hospital. This was to reduce the number of variables associated with using various clinics throughout the healthcare facility. Targeting research to a single clinic, the Outpatient clinic, within the hospital still allowed the study to access a number of providers and a high volume of patients that were willing to participate in the study. It also allowed for obtaining a greater amount of information about the population through the sample to have a better representation of the findings. Even though the results are not generalizable, they still allow the basic structure to ascertain similar issues elsewhere. Future research should focus on conducting similar studies across different Native American clinics. Additional depth can be added by utilizing Quality Function Deployment tools to assess in detail service design and delivery.

In addition, it can be argued that the SQG model incorporates cultural aspects of expectations and needs through the “Past experiences,” “Personal Needs,” and “Word of mouth/Social media.” However, it does not incorporate cultural aspects of values and perceptions. Future research should focus on capturing those in the SQG model and how those can affect the Expected Service for patients and providers’ Service Design.

## Data Availability

All data generated or analyzed during this study are included in this published article at the aggregate level.

## Abbreviations

CAHPS: Consumer Assessment of Healthcare Providers and Systems
CMS: Centers for Medicare & Medicaid Services
HCAHPS: Hospital Consumer Assessment of Healthcare Providers and Systems
IHS: Indian Health Service
PFCC: Patient-and Family-Centered Care
SQG: Service Quality Gap
